# Associations between objective and subjective measures of sleep among rural older adults in the United States

**DOI:** 10.1002/alz70856_103078

**Published:** 2025-12-26

**Authors:** Hye Won Chai, Abigail Stephan, Christy Phillips, Alyssa A. Gamaldo, Charlene Gamaldo, Lesley A. Ross

**Affiliations:** ^1^ Clemson University, Institute for Engaged Aging, Seneca, SC, USA; ^2^ Johns Hopkins School of Medicine, Columbia, MD, USA

## Abstract

**Background:**

Sleep has been linked cognitive function among older adults, and acts as a meaningful predictor of future Alzheimer's Disease and Related Dementias (AD/ADRD); however, there are diverse types of sleep metrics, including daily self‐reports, retrospective surveys, and subjective and objective measures of clinical sleep, which can contribute to inconsistencies across study findings. Examining the associations between different measures of sleep may provide insights to better understand the role of sleep in AD/ADRD risk and prevention. Also, older adults in rural areas who have higher risk of cognitive decline and AD/ADRD have not received much attention in previous sleep literature. Therefore, this study aimed to explore the associations between objective measures of sleep measured via a sleep profiler assessment and self‐reported daily sleep and between subjective, survey‐based Pittsburgh Sleep Quality Index (PSQI) and objective sleep among rural older adults.

**Method:**

We used baseline data from the Everyday Function Intervention Trial (NCT04651582). Sample included participants who completed all three measures of sleep (*N* = 28). Fourteen days of smartphone‐based self‐reported sleep measures included overall means and day‐to‐day variations in sleep quality, difficulty falling asleep, difficulty staying asleep, and feeling refreshed after wake. PSQI included sleep quality, latency, disturbance, duration, efficiency, medication, and daytime dysfunction. Wireless sleep electroencephalography (EEG) monitor (Sleep Profiler™) was used to measure REM percent, NREM Stage 1‐3 percentage, REM latency, and NREM 3 latency.

**Result:**

Linear regression models showed that higher NREM Stage 3 percentage (i.e., longer deep sleep) was associated with better daily sleep quality and feeling more refreshed after waking up every morning. In addition, higher PSQI total score (i.e., worse sleep) was related to longer NREM 3 latency (i.e., longer time until deep sleep stage).

**Conclusion:**

Results suggest that objective measures of NREM sleep were largely in concordance with subjective measures of sleep (i.e., daily reports of sleep and survey‐based PSQI) and that PSQI could be used as a time and cost‐effective measure of sleep quality with similar accuracy to objective measures of sleep among rural older adults who may lack access to sleep study diagnostics, providing opportunities for ADRD prevention through early sleep interventions.

